# Chemometrically Assisted Optimization of Pregabalin Fluorescent Derivatization Reaction with a Novel Xanthone Analogue and Validation of the Method for the Determination of Pregabalin in Bulk via a Plate Reader

**DOI:** 10.3390/molecules27061954

**Published:** 2022-03-17

**Authors:** Nikolaos Kritikos, Aikaterini Iliou, Amalia D. Kalampaliki, Evangelos Gikas, Ioannis K. Kostakis, Benoît Y. Michel, Yannis Dotsikas

**Affiliations:** 1 Laboratory of Pharmaceutical Analysis, Department of Pharmacy, National and Kapodistrian University of Athens, Panepistimioupoli Zografou, GR-157 71 Athens, Greece; n.kritikos.pharm@gmail.com (N.K.); katerinail@pharm.uoa.gr (A.I.); 2 Division of Pharmaceutical Chemistry, Department of Pharmacy, National and Kapodistrian University of Athens, Panepistimioupoli Zografou, GR-157 71 Athens, Greece; akalampa@pharm.uoa.gr (A.D.K.); ikkostakis@pharm.uoa.gr (I.K.K.); 3 Laboratory of Analytical Chemistry, Department of Chemistry, National and Kapodistrian University of Athens, Panepistimioupoli Zografou, GR-157 71 Athens, Greece; vgikas@chem.uoa.gr; 4 Institut de Chimie de Nice, CNRS, UMR 7272, Université Côte d’Azur, Parc Valrose, CEDEX 2, 06108 Nice, France; benoit.michel@univ-cotedazur.fr

**Keywords:** fluorescence, xanthone, derivatization reaction, plate reader, pregabalin, experimental design, central composite design

## Abstract

Quantitation of chromophore-free analytes is always a challenge. To this purpose, derivatization of the analyte constitutes a common strategy, leading to a product with a strong signal. In the current study, a novel xanthone analogue was utilized for the first time for the derivatization of pregabalin, a model analyte with a primary amine moiety that lacks a chromophore. The fact that only the xanthene-based derivative, formed after the derivatization reaction fluoresces, enables avoiding its chromatographic separation from the reagent and thus reducing the analysis time of a series of samples in 1–2 min via a plate reader. The reaction conditions were optimized via a central composite design (CCD), with fluorescence signal as the measure of the yield. The following factors that affect the derivatization reaction were chosen: (a) temperature, (b) reaction time, and (c) triethylamine solution volume used to drive the reaction to completion. After the identification of the optimal conditions, the method was validated according to ICH guidelines, using a fluorescence plate reader for signal measurement (λ_ex_ = 540, λ_em_ = 615 nm). Finally, the newly developed high-throughput method was applied to the determination of drug content in pregabalin bulk.

## 1. Introduction

Pregabalin (or (*S*)-3-(aminoethyl)-5-methyl hexanoic acid) is an antiepileptic drug used for seizure control of epileptic patients [1]. It is also increasingly prescribed for the treatment of neuropathic pain including diabetic peripheral neuropathy and postherpetic neuralgia [1], as well as for generalized anxiety disorder [2] and fibromyalgia [3]. In 2017, pregabalin was one of the top-selling drugs globally, as it is frequently used off-label for other pain disorders [4,5]. There are raised concerns about pregabalin misuse as an “ideal psychotropic drug” [6] with an increased risk of severe adverse effects, which led to its reclassification together with gabapentin to Class C drugs in the UK in April 2019 [7].

Pregabalin is an alkylated analogue of *γ*-aminobutyric acid, the main inhibitory neurotransmitter of the central nervous system. It belongs to the subclass of zwitterionic antiepileptic drugs, which also encompasses vigabatrin and gabapentin. These drugs pose similar chemical structures holding both an amino and a carboxylic group and subsequently similar physicochemical properties [8]. The antiepileptic, analgesic, and anxiolytic actions of pregabalin are mediated through binding to the a2δ subunit of the voltage-sensitive calcium channels, leading to decreased release of excitatory neurotransmitters, including glutamate and norepinephrine (noradrenaline) [9,10].

The assay determination of pregabalin is not officially described in pharmacopoeias, and the development of a suitable method for its determination in bulk or pharmaceutical products is still crucial. Several spectrophotometric and spectrofluorimetric methods have been applied providing rapid and reliable results [8]. However, since pregabalin does not exhibit significant absorption in the UV-Vis range or fluorescence, direct spectrometric methods failed to provide sufficient sensitivity, highlighting the need for a derivatization step.

A variety of derivatization reagents have been used for the fluorescent derivatization of pregabalin, including *π*-acceptors [11,12,13], ninhydrin [11,14], 2,4-dinitrofluorobenzene [13,15], 1,2-naphthoquinone-4-sulfonic acid sodium salt [15,16], among others, for UV-Vis detection, and 7-chloro-4-nitrobenzofurazan [14] and fluorescamine [13,17] for fluorescence sensing. Chromatographic techniques, including liquid chromatography (LC) with UV or fluorescence detection, gas chromatography (GC) as well as high-performance thin-layer chromatography (HPTLC), have also been widely applied. Evaporative light scattering detectors (ELSD) could be an alternative, but little seems to be done in this direction. The few reported GC methods used ethyl chloroformate as the derivatization reagent [18,19,20], while most LC methods included a derivatization step to increase method sensitivity [21,22,23,24,25,26]. LC methods applied without prior derivatization also suffered from elution of the active ingredient close to the dead volume [27,28,29]. On the other hand, HPTLC was performed with densitometric scanning without prior derivatization [30]. A method exploiting generic automated sequential injection for the fluorometric determination of pregabalin and gabapentin in pharmaceutical preparations used on-line derivatization with *o*-phthalaldehyde [31]. Furthermore, capillary electrophoresis and nuclear magnetic resonance (NMR) spectroscopy were exploited for the determination of pregabalin derivatives using different cyclodextrins as chiral selectors [32], while potentiometric methods have also been reported [33,34].

The lack of a chromophore/fluorophore in pregabalin structure requires a fast and straightforward derivatization protocol in most of the applied methods. Due to the high cost of reagents and the large number of factors affecting a derivatization reaction, such as solvent, reagent concentration, reaction time, and molar ratio of the reaction, special attention should be given to the optimization of reaction conditions. In this context, the design of experiments (DoE) plays an essential role as many factors can be examined simultaneously in a predetermined number of experiments [35]. DoE requires less time, effort and resources than the traditional one factor at a time (OFAT) approach, in which a single factor is examined each time while keeping the others constant. DoE facilitates the collection of a large amount of information with a minimum number of experiments, while accounting for putative interactions between the factors examined. Although DoE has been widely used in analytical chemistry, its utility in synthetic chemistry remains largely unknown, especially in the academic field [36]. A few previous studies report significant applications of DoE in synthetic chemistry, leading to improved yields of high purity products in the shortest time and with minimum amounts of reagents [37,38,39].

In a previous study from our laboratories, a novel xanthene-based reagent for the fluorogenic derivatization of primary amines was designed, synthesized and characterized, and then its application to the derivatization of cyclopropylamine was examined [40], with the latter acting a model compound. The reagent is selective for primary amines, as the reaction products with secondary amines exhibit no shift in UV absorption and fluorescence. Taking advantage of the amino group in the pregabalin structure, in this study we utilized this xanthene-based dye for the fluorogenic derivatization of pregabalin and subsequently developed and validated a spectrofluorometric method for its determination in active pharmaceutical ingredient (API) bulk. Moreover, we aimed to optimize the derivatization parameters using a design of experiments (DoE) approach employing a central composite design (CCD) in order to improve the yield of the derivatization reaction with a minimum number of experiments. Overall, the current study suggests a novel and very effective derivatization reagent for the fluorescent determination of primary amines lacking chromophore/fluorophore, such as pregabalin. The fact that only the derivatized analyte fluoresces, constitutes its most significant advantage over the majority of the existing derivatization reagents, as it allows skipping the chromatographic separation from the reagent. On the contrary, the signal is measured directly from the reaction mixture and by utilizing a plate reader the analysis of tens of samples can be completed in a couple of min.

## 2. Materials and Methods

### 2.1. Reagents 

Derivatization reagent (1-bromomethyl-2-nitro-9*H*-xanthene-9-one, **8**) was synthesized in our laboratory [40]. Reference standard of pregabalin was obtained from Pfizer, Inc. (Groton, CT, USA). Methanol (HPLC grade) was obtained from Merck (Athens, Greece), while acetonitrile (LC/MS grade) was purchased from Carlo Erba Reagents (Darmstad, Germany). Triethylamine was obtained from Sigma–Aldrich (St. Louis, MO, USA). Aqueous solutions were prepared with de-ionized and double-distilled water (resistivity > 18 MΩ) from Merck Millipore (Darmstad, Germany).

### 2.2. Synthesis of the Novel Derivatization Reagent

**Figure 1:**
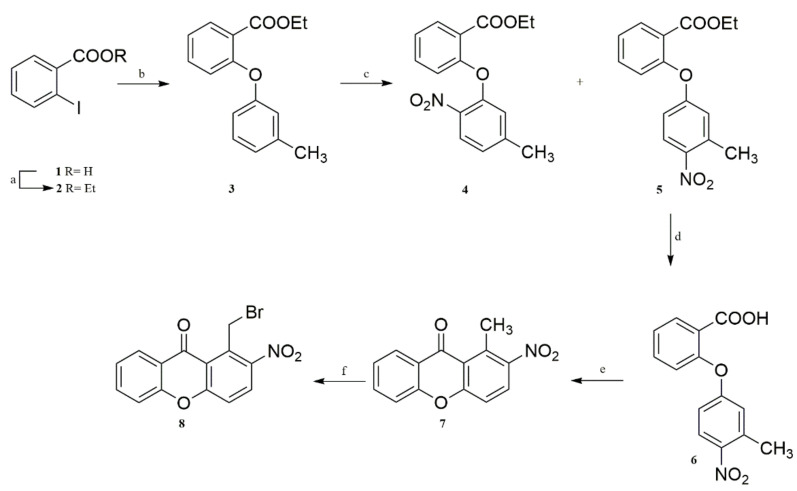
Synthesis of the derivatization reagent (**8**): Reagents and conditions: a: conc. H_2_SO_4_, abs. ethanol, reflux; b: m-cresol, CuCl, K_2_CO_3_, pyridine, reflux; c: fum. HNO_3_, (CH_3_CO)_2_O, 24 h, 0 °C; d: 40 % NaOH, ethanol, rt; e: PPA, 100 °C; f: NBS, dibenzoyl peroxide, CCl_4_, UV (80-W lamp), 8 h.

The synthesis of the xanthene-based derivatization reagent **8**, is depicted in Figure 1. Commercially available 2-iodobenzoic acid (**1**) was esterified to the corresponding ethyl ester **2**, which upon reaction with m-cresol, via Ullmann conditions, afforded ester **3**. Treatment of compound **3** with fuming nitric acid in the presence of acetic anhydride resulted in a mixture of the nitro compounds **4** and **5**, which were separated by column chromatography. Ester **5** was then saponified under mild conditions, and the resulting carboxylic acid **6** was ring-closed upon treatment with polyphosphoric acid (PPA) providing xanthone **7**, which was unambiguously identified by 1- and 2D-NMR experiments. The target derivatization reagent **8** was obtained from xanthone **7** upon treatment with *N*-bromosuccinimide (NBS) via UV irradiation.

### 2.3. Instrumentation

The derivatization reaction took place in Thermo Scientific Reacti-Therm Heating Module (Metrolab, Athens, Greece). All measurements were performed with a Fluostar Galaxy multifunctional microplate reader (BMG LabTechnologies GmbH, Germany). Fluorescence optics were installed, and the excitation and emission wavelengths were set at 540 and 615 nm, respectively, by selecting the relevant filters. The 96-well black polystyrene plates and 1.5-mL Eppendorf tubes were obtained from Nunc (Pnoi, Athens, Greece).

An Accela UHPLC system (Thermo Fisher Scientific, Bremen, Germany) equipped with a binary pump and an autosampler was utilized to separate the excess of derivatization agent from the product on an Ascentis Fused Core C18 column (100 × 2.1 mm, 2.7 μm). Mass spectra were recorded on a hybrid LTQ™ Orbitrap Discovery XL instrument (Thermo Fisher Scientific), enabling the characterization of the reaction product. The whole system was controlled by the Xcalibur 2.1 software.

### 2.4. Solutions

Pregabalin stock solution (200 μg/mL) was prepared in MeOH. Calibration standards in ACN obtained from this stock solution were as follows: 0.500, 1.00, 2.00, 5.00, and 10.0 μg/mL. All standards were prepared fresh on a daily basis. 

Xanthone **8** stock solution (1 mg/mL) was prepared in ACN and 10% triethylamine solution was prepared by diluting triethylamine with ACN (1:10 *v*:*v*).

The reaction mixture used for the experimental design had a total volume of 1 mL and was prepared in 1.5-mL Eppendorf tubes. The mixture was composed of 40 μL of pregabalin working solution, 200 μL of xanthone **8** stock solution and the remaining volume was completed with a 10% triethylamine solution and ACN the amounts of which depends on the values suggested by the runs of the experimental design. At the end of the reaction in the Thermo heating block, 200 μL of the mixture was transferred with a micropipette into the respective well of the 96-well plate.

### 2.5. Software

The central composite design was created by Design-Expert v.10-trial version (Stat-Ease-Inc, Minneapolis, MI, USA) and MS Office Excel 2013 (Microsoft, Redmond, Washington, DC, USA) was utilized for statistical evaluation of the obtained data.

## 3. Results and Discussion

### 3.1. Optimization of the Reaction Conditions

**Figure 2:**
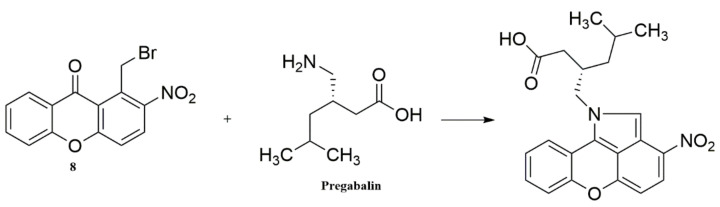
Derivatization reaction of pregabalin with the novel xanthone analogue.

Preliminary experiments were carried out to screen for potential factors that could influence the reaction yield, and they were evaluated by the derivatized pregabalin’s emission signal (610 nm). Initially, the formation of the derivatization product of pregabalin (Figure 2) could be confirmed by the appearance of purple color. Based on these experiments, the following factors were selected: (A) temperature, (B) reaction time, and (C) added volume of 10% triethylamine solution. The latter was used as a reagent that could create basic conditions to the reaction mixture, in order to scavenge the generated HBr and shift the reaction to completion. As mentioned above, acetonitrile was selected as the solvent of the reaction mixture, providing a polar environment that promotes nucleophilic substitution reactions, without participating in the reaction mechanism, which could potentially happen with a protic solvent such as methanol. It should be also noted that the derivatization reagent was added in a high molecular excess (>10/1) with the aim of obtaining a higher reaction yield, expressed as fluorescence signal (response).

The next step was the selection of the appropriate experimental design that will define the appropriate experiments to be performed in the experimental region of interest. To this purpose, CCD was chosen as the most suitable, having a full factorial design and an a ± 1.68 (α) star design with four replicates in the central point. That way all variables are examined at five levels. Table 1 presents the plan of 18 experiments obtained via CCD, along with the resulting responses. Experimentally obtained response values were transformed by a logarithmic function before modeling.

**Figure 3:**
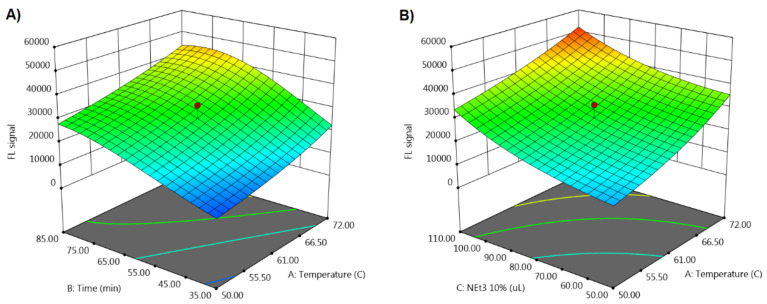
3D graphs: (**A**) FL_signal_ = f (time, temperature) and (**B**) FL_signal_ = f (Et_3_N 10%, temperature).

The modeling was conducted by Design-Expert v.10-trial version and for the response FL_signal_ a quadratic model was suggested (Figure 3). Its calculated coefficients for coded factor values are given in Equation (1). The adequacy of the obtained model was confirmed by ANOVA (*p* = 0.0001), lack of fit test (*p* = 0.19), R^2^ (0.96), adj. R^2^ (0.91), and pred. R^2^ (0.73) values. Statistically significant coefficients are marked with two asterisks for *p* < 0.01, and three asterisks for *p* < 0.001.

Ln(FL_signal_) = 10.22 + 0.35*** × A + 0.46*** × B + 0.32*** × C − 0.13 × A × B − 0.13 × A × C − 0.15 × B × C + 0.014 × A^2^ − 0.25** × B^2^ + 0.071 × C^2^ (1)

All factors were proven to have a statistically significant positive effect on FL_signal_, meaning that increasing their values causes enhancement of the reaction yield. The existence of a single response simplifies the procedure to reach the optimal conditions of the factors that result in maximization of the signal. To this purpose, Derringer’s desirability function was utilized [41]. A series of solutions with excellent desirability value (D ≥ 0.998) was obtained by adjusting the importance coefficients, weights, and range of the response, according to the defined objective. They all had very similar values, i.e., in the range 70.7–72 °C for temperature, 65.7–70.3 min for time, and 107–110 μL for the volume of 10% Et_3_N solution. The latter indicates that there is probably an optimal region (design space) and not just a single solution.

From the obtained excellent solutions, the following values per factor were selected as optimal (D = 0.999): temperature 72 °C, time 68.8 min, and Et_3_N 10% solution of 110 μL. The predicted value of the response (52,645.2 RFU) was in excellent agreement with the experimentally obtained value (53,527 RFU) via the plate reader, confirming the predictive abilities of the defined model, as well as the efficiency of the optimal conditions obtained.

### 3.2. Chromatographic and Mass Spectrometry Analysis

**Figure 4:**
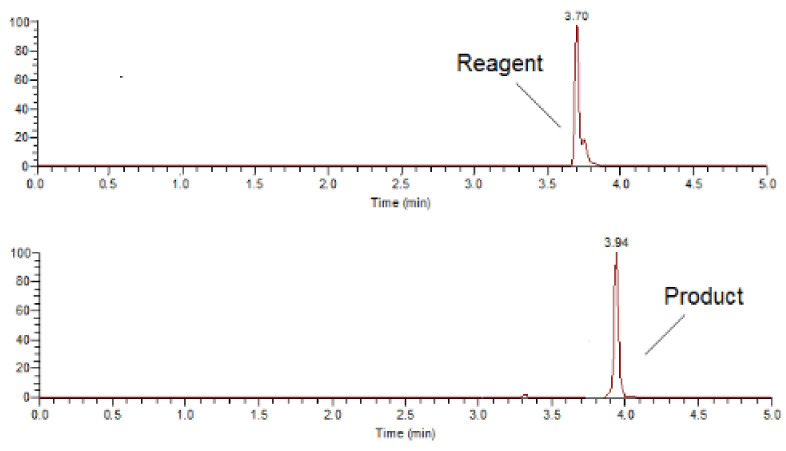
Representative chromatograms of the derivatization reagent (**top**) and the derivatized pregabalin (**bottom**).

**Figure 5:**
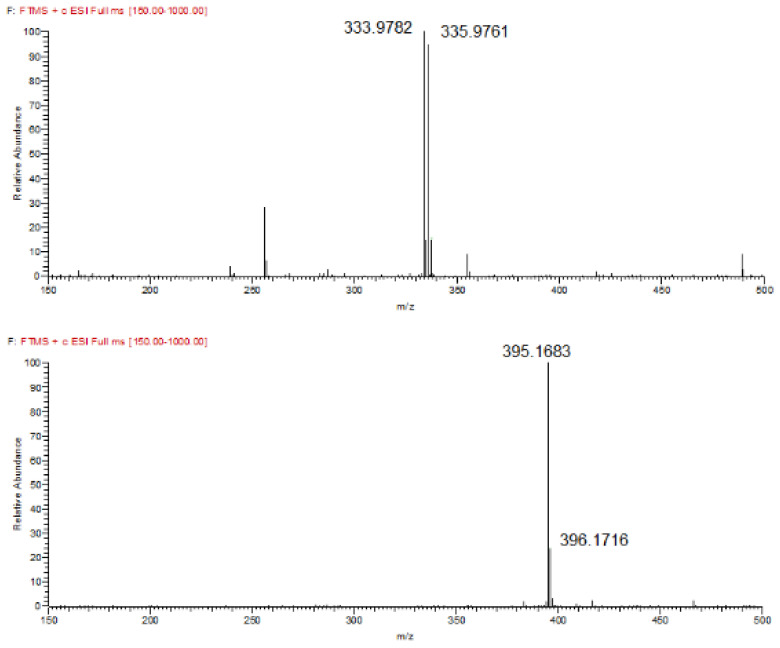
MS spectra showing the peaks assigned to the derivatization reagent (**top**) and derivatized pregabalin (**bottom**).

In order to confirm the formation of the derivatization product following the abovementioned optimal conditions, a UHPLC-MS method was utilized. Initially, our aim was to separate the product from the excess of derivatization reagent by applying a gradient elution program [40]. The chromatograms presented in Figure 4 demonstrate that a baseline separation was achieved. Both analytes were precisely identified in the presented MS spectra (Figure 5) obtained from the respective chromatographic peaks. It should be stated that apart from the identification via *m*/*z* values, the presence of the characteristic couple of the two molecular ion isotopes with similar abundance (top spectrum) attributed to bromine in the reagent structure and its absence in the derivatized pregabalin (bottom spectrum) where this halogen is not present, is another piece of evidence of the abovementioned reaction.

### 3.3. Stability of the Fluorescence Signal

**Figure 6:**
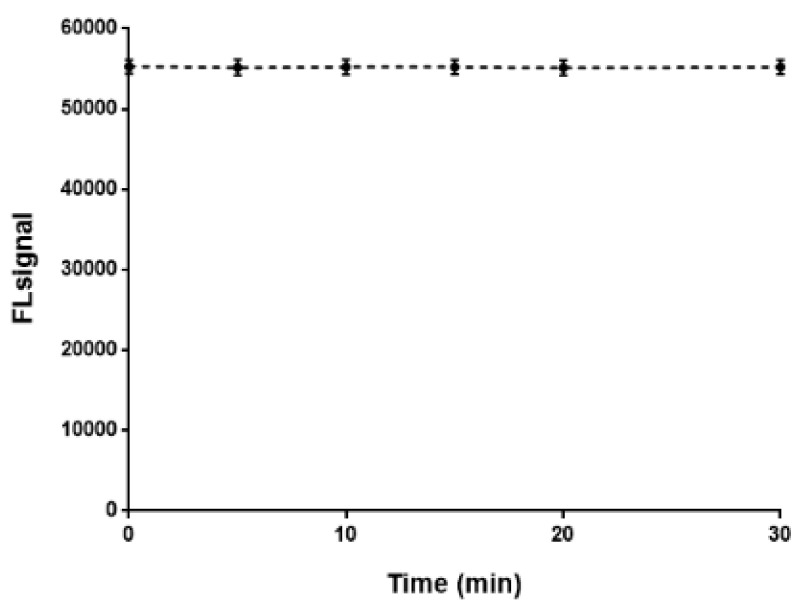
Fluorescence signal intensity vs. time over a 30 min period (*n* = 3).

A critical parameter of this method that can affect its applicability is the stability of the fluorescence signal. The current approach enables the simultaneous conduction of a series of reactions in the heating block followed by the transfer in the 96-well plate. For 30 samples, which was the maximum number/plate reached in the current assay, a time period of 12 min is adequate for the transfer of the reaction mixture, while the duration of the measurement of all samples in the plate reader was less than 1 min. In order to take into account any delay in the procedure, signal kinetics was studied for a time period of 30 min. As shown in Figure 6, the signal is essentially stable, allowing reliable measurements to be made within this period. It is prevalent that a different period for signal stability should be tested for other analytical techniques (i.e., chromatography).

### 3.4. Method Validation

The method was validated for the determination of pregabalin in bulk according to ICH guidelines [42] under the optimal reaction conditions. The validation procedures included specificity, linearity, accuracy, precision, stability, and robustness.

#### 3.4.1. Specificity

The derivatization reaction is specific for primary amino groups. In the case of the determination of API in bulk no interference (signal) was observed, as pregabalin is replaced by ACN. However, the excipients of Lyrica^®^ hard capsules content (lactose monohydrate 75, maize starch 7.5, talc 1.5 mg) were available and therefore an additional specificity test was performed. Again, no signal was obtained in the presence of the excipients, since they do not possess amino groups in their structure. The current test proves that the method is suitable, in terms of specificity, for the determination of pregabalin in hard capsules.

#### 3.4.2. Linearity

The calibration curve was obtained by plotting the signal intensity versus the concentration of the standard solutions. A linear regression equation was obtained (slope: 6020 ± 8, intercept: 15.46 ± 41.94) over the range 0.500–10.0 μg/mL with a coefficient of determination R^2^ = 0.999.

#### 3.4.3. Limit of Detection (LOD) and Limit of Quantification (LOQ)

The LOD of the method was defined as the back calculated concentration of pregabalin that corresponds to the mean signal of 6 zero samples plus 3 times the standard deviation (SD) of these samples. At the same time, the LOQ was estimated as the sum of the mean signal of 6 zero samples plus 10 × SD. The concentrations 0.0770 and 0.237 μg/mL were estimated as LOD and LOQ of the method, respectively. These values are comparable to relevant obtained by other derivatization reagents.

#### 3.4.4. Accuracy

Accuracy was estimated as the percentage of analyte recovered by the assay (%R), following the analysis of the aforementioned mixtures. In this study, the following levels of pregabalin were used in triplicate: 4.00 (80%), 5.00 (100% reference level), and 6.00 μg/mL (120%). The requirements of a mean recovery of 98% ≤ %R ≤ 102% for each level, as well as of 98% ≤ %R ≤ 102% between the three levels were fulfilled, as the obtained range of %R was 98.82–100.3 (Table 2). In addition, the criterion %RSD ≤ 1 was met (%RSD ≤ 0.82).

#### 3.4.5. Precision

Repeatability and intermediate precision were assessed in the current method by using six replicate spiked samples at the 100% concentration level (5.00 μg/mL), in three consecutive days. The criterion of %RSD ≤ 2, related to the percentage recovery of the measurements, was achieved, proving the method to be precise during different days. Table 3 presents the %Recovery for six replicate spiked samples and the %RSD values for repeatability (%RSD_r_). As for the intermediate precision, the value of %RSD_R_ 0.79 was estimated.

#### 3.4.6. Stability

The stability of the standard (underivatized) solutions was evaluated in triplicate over a period of 2 days (0, 5, 24, and 48 h) and under different storage conditions (25 °C and 5 °C). The tests revealed that the standard solutions remained stable for 48 h, under both ambient and refrigerated conditions. The %RSD values of each standard solution were assessed and found to be ≤0.85.

#### 3.4.7. Robustness Testing

The robustness of the analytical method was estimated by performing deliberate slight variations in the derivatization reaction conditions around the nominal level. Results are summarized in Table 4 and indicate that these slight changes did not result in significant variations in the obtained values and confirmed that the current assay was robust.

## 4. Conclusions

A simple, robust and sensitive spectrofluorometric method for the determination of pregabalin in bulk was developed and validated, using a novel xanthone analogue as a derivatization agent. The novel agent that reacts with primary amines had no fluorescence properties, while the derivatized pregabalin had a strong vermilion signal. The latter avoids the need for a chromatographic technique and, therefore, all measurements can take place in a plate reader allowing their completion in seconds. The reaction conditions were optimized by applying a central composite experimental design. The method was found to be specific, linear, precise, accurate, and robust throughout the concentration range investigated. The assay demonstrated to be suitable for the determination of pregabalin in its API bulk but, since the excipients in pregabalin hard capsules do not bear primary amines, it can be transferred for the determination of the drug in pharmaceutical dosage forms. Moreover, the current protocol can be utilized to the determination of other drugs with similar properties.

## Figures and Tables

**Table 1:** Plan of experiments for CCD and experimentally obtained responses. Coded values for factors are presented in parentheses.

	Factors			Response
	A: Temperature (°C)	B: Time (min)	C: 10% Et_3_N (μL)	FL Signal
1	61.00 (0)	60.00 (0)	80.00 (0)	26,080
2	61.00 (0)	60.00 (0)	80.00 (0)	26,490
3	79.50 (+a)	60.00 (0)	80.00 (0)	46,600
4	42.50 (−a)	60.00 (0)	80.00 (0)	13,920
5	61.00 (0)	102.04 (+a)	80.00 (0)	27,960
6	61.00 (0)	60.00 (0)	29.55 (−a)	20,910
7	50.00 (−1)	35.00 (−1)	110.00 (+1)	20,350
8	72.00 (+1)	85.00 (+1)	50.00 (−1)	42,810
9	72.00 (+1)	35.00 (−1)	50.00 (−1)	17,280
10	61.00 (0)	60.00 (0)	130.45 (+a)	42,690
11	50.00 (−1)	85.00 (+1)	50.00 (−1)	21,280
12	61.00 (0)	60.00 (0)	80.00 (0)	25,280
13	72.00 (+1)	35.00 (−1)	110.00 (+1)	39,720
14	50.00 (−1)	35.00 (−1)	50.00 (−1)	5150
15	72.00 (+1)	85.00 (+1)	110.00 (+1)	54,370
16	50.00 (−1)	85.00 (+1)	110.00 (+1)	45,850
17	61.00 (0)	17.96 (−a)	80.00 (0)	5240
18	61.00 (0)	60.00 (0)	80.00 (0)	33,570

**Table 2:** Accuracy results.

	80% Level	100% Level	120% Level
	100.5	99.77	98.25
	100.1	100.9	99.05
	98.92	100.2	99.16
**Mean**	99.84	100.3	98.82
**SD**	0.82	0.57	0.50
**%RSD**	0.82	0.57	0.50

**Table 3:** Results of repeatability for 3 days (*n* = 6).

Compound	Day 1	Day 2	Day 3
Pregabalin	100.32	99.08	101.33
	101.27	101.25	100.62
	100.44 101.28 100.39 99.12	100.60 101.28 100.25 100.78	101.39 101.32 102.14 99.88
**%RSD**	**0.79**	**0.81**	**0.76**

**Table 4:** Robustness testing results of 100% concentration level (5 μg/mL).

Parameter	%Recovery *	%RSD
**Temperature (°C)**		
71	101.80	0.75
72	100.25	
73	100.27	
**Time (min)**		
67.8	100.45	0.82
68.8	99.95	
69.8	101.77	
**10%Et_3_N (μL)**		
108	100.58	0.87
110	101.23	
112	99.57	

* Mean values (*n* = 3).

## Data Availability

Data are contained within the article.

## Sample Availability

Samples of the compounds are available from the authors.
